# Kidney biopsy in diabetic kidney disease. Yes, but in very selected cases

**DOI:** 10.1093/ckj/sfad268

**Published:** 2023-10-26

**Authors:** Carmine Zoccali

**Affiliations:** Renal Research Institute, New York, NY, USA; Istituto di Biologia molecolare e genetica (BIOGEM), Ariano Irpino (Avellino), Italy; Associazione Ipertensione Nefrologia e Trapianto Renale (IPNET) c/o Nephrology, Transplantation and Hypertension Unit, Ospedali Riuniti, Reggio Cal, Italy

**Keywords:** diabetic kidney disease, diabetic nephropathy, kidney biopsy

## Abstract

The debate on kidney biopsy in diabetic kidney disease (DKD) is multifaceted. Loreto Gesualdo and colleagues argue for its broader application, claiming that biopsies can offer precise diagnostic data and guide personalized treatment plans. On the other hand, Alberto Ortiz opposes this, citing insufficient evidence, resource constraints and potential risks. He suggests that alternative diagnostic methods, such as advanced imaging techniques and serological markers, will obviate the need for biopsy in most patients. Both sides agree on the need for individualized patient care, and open discussions between healthcare providers and patients about the procedure's risks and benefits. The application of kidney biopsy in these patients needs to consider clinical evidence, practical limitations and patient preferences, and demands a balanced, case-by-case approach. Overall, this moderator believes that, although fundamental in clinical research, kidney biopsy in DKD is infrequently needed.

The use of kidney biopsy in diabetic patients with evidence of kidney dysfunction/damage is almost ignored in current guidelines. Opinions and approaches to diagnosing kidney disease in people with diabetes vary. In this controversy, Loreto Gesualdo, Marco Fiorentino, Francesca Conserva and Paola Pontrelli [1] argue in favour of broadening the indication for kidney biopsy in patients with diabetes. They believe that the heterogeneous nature of renal complications in type 2 diabetes mellitus (T2DM) calls for a more individualized approach to patient care, where kidney biopsy can guide therapeutic decisions and pave the way for innovative, non-invasive diagnostic methods. They point out that 20-year-old, groundbreaking studies identified three renal damage classes in T2DM patients: classical diabetic glomerulosclerosis, vascular and ischemic glomerular changes, and other glomerulonephritides [2]. These authors argue that the shift from a generic approach to personalized medicine is underscored by the wealth of information that can be gleaned from renal biopsies.

Alberto Ortiz [3] presents several detailed arguments against extending the use of kidney biopsy. Firstly, he notes that there is insufficient evidence to support the use of kidney biopsy in people with diabetes. While kidney biopsy can help identify non-diabetic nephropathies that may require different treatment approaches, the majority of cases of diabetic kidney disease (DKD) can be diagnosed and treated without biopsy. Secondly, he remarks that expanding the use of biopsy to all people with diabetes with severe chronic kidney disease could lead to a significant increase in the number of biopsies performed and, therefore, in the number of complications, including the risk of nephrectomy and death. Expanding the use of biopsy would require significant resources, including in-patient facilities and expert pathologists, which may not be available in many health contexts. Alberto Ortiz suggests that non-invasive imaging techniques, such as novel ultrasound and magnetic resonance imaging, can be used to identify renal structural abnormalities (including an estimate of the degree of fibrosis) [4]. On the other hand, urinary albumin-to-creatinine ratio and serum creatinine remain time-honored and valid biomarkers to assess kidney function and damage, which are helpful to identify patients at risk of progression to end-stage renal disease.

## MODERATOR CONSIDERATIONS

A balanced viewpoint on using kidney biopsy for clinical decision-making in patients with DKD should consider both the potential benefits and the drawbacks of the procedure (Table 1).

**Table 1:** This table outlines the pros and cons of using kidney biopsy in the diagnosis and management of DKD. As per the information, individualized patient assessment and open discussions between healthcare providers and patients are vital for making an informed decision on whether to proceed with a kidney biopsy.

Method	Pros	Cons
Kidney biopsy in DKD	1. Definitive diagnosis	1. Invasive and potential risks
	2. Identifies atypical features	2. Resource-intensive (cost, time, expertise)
	3. Differentiates from other systemic conditions	3. Lack of comparative clinical trials
	4. Crucial for immunotherapy in specific cases	4. May not be suitable for older patients or those with multiple comorbidities
	5. Benefits broader medical research	5. Some patients may be reluctant due to potential complications

There is still no clinical trial comparing clinical outcomes among policies guided by kidney biopsy and policies driven by standard kidney dysfunction and damage biomarkers. While the procedure can provide valuable diagnostic and prognostic information, it comes with potential risks and costs. The benefits must be weighed against the risks in each patient's clinical scenario. In most cases, non-invasive tests might provide sufficient information for clinical management. Collaboration between nephrologists and patients, with open discussion about the pros and cons of the procedure, is vital in making an informed decision. Pragmatic trials testing the usefulness of kidney biopsy in DKD remain an unmet clinical need.

Individualized decision-making can be guided by the presence of atypical features, including sudden-onset proteinuria, active urinary sediment (e.g. hematuria with red cell casts), or rapid decline in kidney function. In the presence of these alterations, all atypical in DKD, a kidney biopsy can be proposed to the patient. In these cases, the suspicion of non-diabetic renal disease would be reinforced by a short duration of diabetes (<5 years) or the absence of retinopathy. Suppose a patient has unexplained low complement levels, positive anti-nuclear antibodies or anti-dsDNA, or other serologic markers suggestive of a systemic disease. In that case, a biopsy can help differentiate primary DKD from other systemic conditions affecting the kidneys. If a non-diabetic glomerular disease is suspected that might benefit from immunosuppression, a biopsy becomes crucial. Older patients or those with multiple comorbidities might be at higher risk for biopsy-related complications. In such cases, the risk–benefit ratio might tilt against a biopsy unless biopsy is deemed essential. The patient's willingness, concerns about potential complications, and understanding of the procedure's benefits and risks are vital. Some patients prefer a definitive diagnosis via biopsy, while others might opt against an invasive procedure.

Finally, biopsies might be done as part of research protocols to understand DKD pathophysiology better. In such cases, the potential benefits of broader medical knowledge are weighed against the risks to the individual patient.

In summary, individualizing kidney biopsy in DKD involves thoroughly evaluating the patient's clinical presentation, potential benefits from the procedure, inherent risks and patient preferences. Collaboration between healthcare providers and patients is crucial, with a clear discussion about the potential pros and cons.
